# Interactive Tooth Separation from Dental Model Using Segmentation Field

**DOI:** 10.1371/journal.pone.0161159

**Published:** 2016-08-17

**Authors:** Zhongyi Li, Hao Wang

**Affiliations:** School of Hydropower and Information Engineering, Huazhong University of Science and Technology, Wuhan, Hubei Province, China; University of Brescia, ITALY

## Abstract

Tooth segmentation on dental model is an essential step of computer-aided-design systems for orthodontic virtual treatment planning. However, fast and accurate identifying cutting boundary to separate teeth from dental model still remains a challenge, due to various geometrical shapes of teeth, complex tooth arrangements, different dental model qualities, and varying degrees of crowding problems. Most segmentation approaches presented before are not able to achieve a balance between fine segmentation results and simple operating procedures with less time consumption. In this article, we present a novel, effective and efficient framework that achieves tooth segmentation based on a segmentation field, which is solved by a linear system defined by a discrete Laplace-Beltrami operator with Dirichlet boundary conditions. A set of contour lines are sampled from the smooth scalar field, and candidate cutting boundaries can be detected from concave regions with large variations of field data. The sensitivity to concave seams of the segmentation field facilitates effective tooth partition, as well as avoids obtaining appropriate curvature threshold value, which is unreliable in some case. Our tooth segmentation algorithm is robust to dental models with low quality, as well as is effective to dental models with different levels of crowding problems. The experiments, including segmentation tests of varying dental models with different complexity, experiments on dental meshes with different modeling resolutions and surface noises and comparison between our method and the morphologic skeleton segmentation method are conducted, thus demonstrating the effectiveness of our method.

## Introduction

In recent years, orthodontic computer-aided-design (CAD) systems are widely used by clinicians to prepare their orthodontics surgery. These CAD systems exploit hardware-supported computer graphics technology to effectively and efficiently plan diagnosis traditionally done manually. These systems provide information precise enough to be used in diagnosis and prognosis, thus free clinical dentists from repeated works and facilitate accurate treatment planning [1–4].

As well as in traditionally case, after acquiring digital dental model which is generated from scanning teeth of patient, dentist firstly need to accurately separate teeth from the digitized dental model in computer-aided orthodontics. After the process of teeth separation is finished, or the exact position of each tooth is determined, dentist is able to measure orthodontic features, to simulate orthodontic procedures and to work out an applicable treatment plan. Therefore, acquiring fine segmentation results of dental models is a critical step to achieving the following diagnostic work.

However, such segmentation work in dental models remains a difficult task. On the one hand, general purpose mesh segmentation approaches are not directly suited for segmenting dental meshes because of special geometrical shapes of teeth and of complex teeth arrangements on dental models.On the other hand, other segmentation approaches proposed to handle digital dental models have some shortcomings, such as tedious, time-consuming and not sufficiently accurate.

There has been extensive study of variation in general mesh segmentation. The negative minima rule proposed by Hoffman et al. [5, 6], states that objects are segmented by human perception at regions of concavity. Based on such common criterion, many existing mesh segmentation methods are proposed, including the K-mean clustering [7], graph cut-based fuzzy clustering [8], random walk algorithm [9, 10], the primitive-fitting-based [11] and spectral analysis methods [12]. These methods, directly seek a single segmentation solution without considering or reusing other potential segmentation solutions, usually do not suffice to generate high quality segmentation results for all kinds of models. In contrast, there are other alternative segmentation methods using more complex geometrical information, like shape diameter function [13], tubular analysis [14] and skeleton-based methods [15]. These methods, however, involve complex implementation procedures and expensive computation costs.

Recently, a smooth scalar or vector field—harmonic field, through computing harmonic functions [16] on mesh, have been introduced for mesh segmentation [17–21]. Compare with other segmentation methods mentioned above, these harmonic field methods, including fully automatic methods and interactive methods, can directly extract concave regions, and the relevant segmentation processes are more efficient and scalable.

When dealing with dental models, the general purpose segmentation methods should be adjusted and specialized, according to complex geometrical shapes and characteristics of dental models. These dental mesh segmentation methods can be classified into two categories, curvature field based method and image based method.

To the curvature field based method, the feature regions that contain potential teeth boundaries are needed to extract by mean curvature thresholding [22, 23] or minimum rule [24–27]. After feature regions are extracted, morphologic skeleton extraction technique [23, 24, 26] are applied to refine these coarse boundaries into strict single-vertex-width teeth boundaries. Other methods, like the flood-fill method [27], the fast marching watersheds method [25] and the snake-based method [22], also directly exploit the feature regions. However, the drawbacks of the curvature field based method are obvious. The curvature field is unreliable due to its sensitivity to noise which is inevitable to digital dental models. More importantly, the selection of threshold value is critical to final segmentation result. Inappropriate threshold value would leads to under-segmentation or over-segmentation, and automatically obtaining appropriate threshold value [28] is at the cost of inaccurate segmentation result with less iteration times, or of expensive computation time with more iteration times.

Given that tooth boundaries are clear in projected 2D images, many authors [29–31] used a specifically designed mesh representation that maps 3D vertices onto 2D vertices and exploits 2D image segmentation techniques to segment dental models. Kondo et al. [30] proposed a highly automatic segmentation method by extracting interstice points on planar and panoramic range images. Grzegorzek et al. [31] applied multiple parallel-range map images to obtain 2D contours and to cut adjacent teeth by connecting significant non-convex points on them. The image based segmentation method, however, lacks three dimensional geometrical information, complex interstices or non-convex points are difficult to extract, which leads to inaccurate cutting between adjacent teeth.

In the article, we proposed a novel, effective and efficient teeth segmentation method based on segmentation field. The segmentation field is a scalar-valued field, which is solved by a linear system defined by a discrete Laplace-Beltrami operator with Dirichlet boundary conditions, which are imposed at a set of constraint points. A concavity-sensitive weighting scheme is applied to calculation of segmentation field, such scheme make the segmentation field exhibits significant greater variation at concave regions of dental mesh.

Through identifying maximum concavity variation, the final segmentation boundaries can be extracted from these contour lines that are sampled from segmentation field.

Overall, the aims and contributions of our dental segmentation method are included as follows:

- Compare to other common Laplacian weighting schemes, the adjusted weighting scheme is introduced to partition teeth from dental models accurately and efficiently.
- A strategy to assign the constraint points is proposed, according to different geometrical shapes of teeth.
- An easy-to-use interactive tool is developed to segment dental models with minimal user interactions.

## Materials and Methods

**Fig 1:**
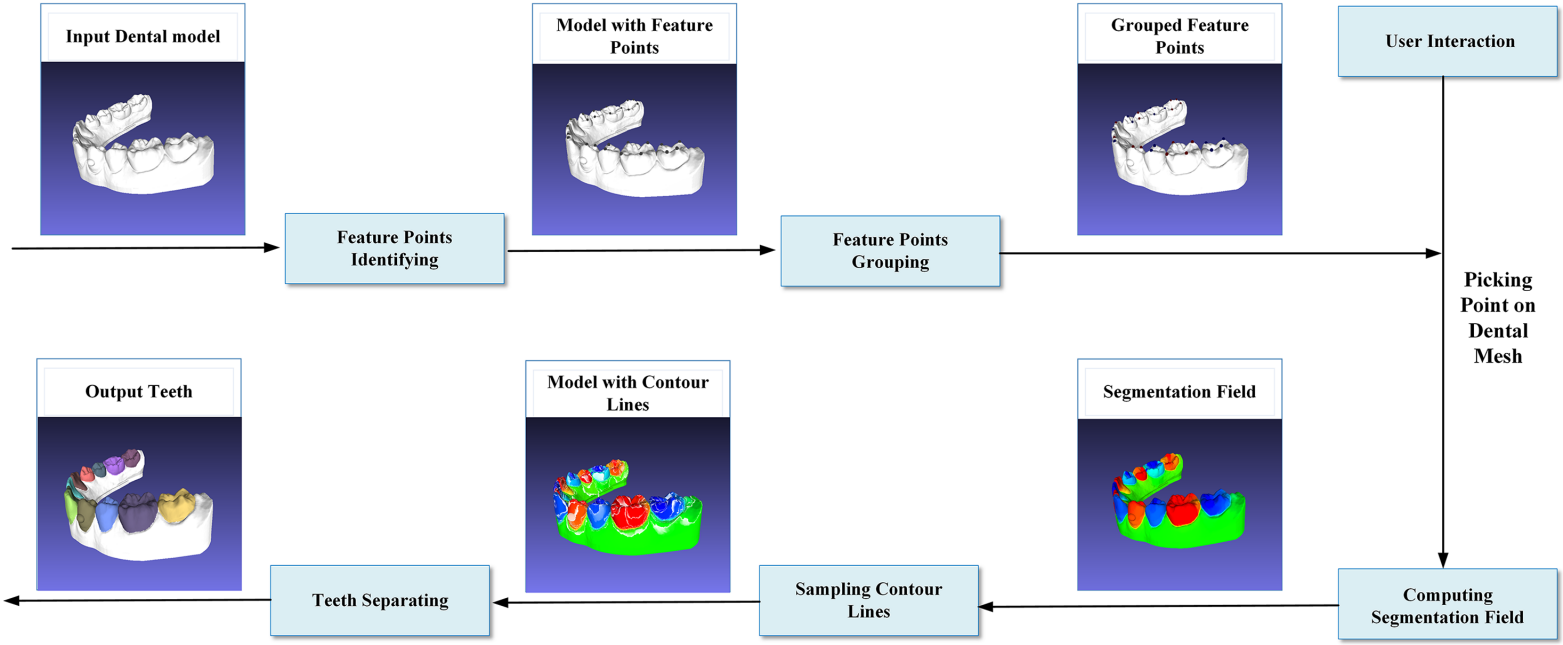
The proposed pipeline for dental mesh segmentation.

A series of strategies are applied by us to accomplish the purposes mentioned in previous section, the overview of our framework as illustrated in Fig 1. The scanned dental model is firstly preprocessed to identify and to group the feature points in dental mesh. These feature points, are assigned to segmentation field as boundary constraints, have the ability to indicate useful geometry information of dental model for tooth segmentation. Through user interaction, after this, a point on mesh is clicked to indicate which tooth the user want to separate from dental mesh, thus the target boundary constraints and the background boundary constraints are assigned according to the relevant location of the clicked point. Then, the segmentation field, whose scalar values are colored from red to blue, is computed based on the concavity-aware weighting scheme. Afterwards, a set of contour lines are sampled from the segmentation field, and the cutting boundaries between tooth-tooth and tooth-gingiva are detected from these contour lines in the concave regions. Finally, all the teeth are separated and colored from the input dental mesh based on the cutting boundaries.

### Definition of the segmentation field

The segmentation field is actually a harmonic field which has been exploited to mesh segmentation [19–21, 32]. In our method, partitioning tooth from dental model relies on these desirable properties of segmentation field. The segmentation field is a smooth scalar field and is so sensitive to concave regions that tooth boundaries can easily extracted from the contour lines which lie on concave regions.

Let *M* = (*V*, *E*) be the given dental model, where *V* is the set of vertex indices, *E* is the set of edges and *v*_*i*_ the location of vertex *i* in space. A segmentation field can be denoted by **u**, a solution to the Poisson equation Δ**u** = 0, which is subjected to Dirichlet boundary conditions imposed by sites *S*, which can be denoted by *S* ∈ {1, 2, …, *n*}. Δ is the Laplace-Beltrami operator, which can be discretized and resulted in a symmetric Laplacian matrix. Such symmetric operator, which firstly presented by Pinkall and Polthier [33], leads to a symmetric and positive-definite linear system, allowing for fast Cholesky factorization. The site constraints are assigned in segmentation field computation, which can be described as

$$u\left(i\right)=u_{i}=s_{i},$$ (1)

where *s*_*i*_ is the prescribed value of segmentation field at site *i*. To obtain constrained segmentation field, we minimize the membrane energy [34]

$$u=arg\min_{x}{\left(||\text{Lx}|\right|}^{2}+\left|\right|P^{1/2}{\left(x-b\right)||}^{2}),$$ (2)

leading to the linear system

(L+P)u=Pb, (3)

where **L** is the concavity-aware Laplacian matrix, **P** is the diagonal penalty matrix

L=D-W, (4)

$$vL_{ij}=\{\begin{array}{cc} \sum_{i}\omega_{ij}, & i=j\\ -\omega_{ij}, & i,j\in E,\\ \;0, & \text{otherwise} \end{array}$$ (5)

$$P_{ij}=\left\{\begin{array}{cc} \alpha , & i\in S,i=j\\ 0, & \text{otherwise}, \end{array}\right.$$ (6)

$$b_{ij}=\left\{\begin{array}{cc} s_{i}, & i\in S\\ 0, & i\notin S. \end{array}\right.$$ (7)

The penalty factor *α* is a large constant used to tweak the importance of constraint satisfaction (*α* = 10^8^ in our experiments), **D** is a diagonal matrix of the row sums of **W**, **W** is the weight matrix which is defined by *ω*_*ij*_, and the selection of weighting scheme and scalar weight assigned to *ω*_*ij*_ are discussed in the following subsection.

### Selection of weighting scheme in segmentation field

Harmonic fields with different Laplacian weighting schemes have been introduced for different application purposes, such as construction of reduced deformation models [35], transformation propagation [36] and shape approximation [37]. However, for the harmonic field that is suited for dental mesh segmentation, there are two conditions should be satisfied as follows:

- The segmentation field should be sufficiently smooth so that the smooth contour lines can be extracted from such field without post-processing, this means that the cutting boundaries to separate target teeth from dental model are clear and can be directly and easily identified.
- Considering the segmentation purpose for dental models, the segmentation field is able to identify the shape variation and is sensitive to concavity regions of models where the candidate cutting boundaries lie on.

To fulfill the conditions mentioned above, the small prescribed values are assigned on the edges lying on concave regions, which leads to the scalar values of segmentation field change abruptly along concave regions, through setting the concavity-aware weighting scheme as

$$\omega_{ij}=\left\{\begin{array}{cc} \frac{|e_{ij}|\cdot \beta }{|G_{i}+G_{j}|+\gamma }, & \text{vertex}\;i\;\text{or}\;j\;\text{is}\;\text{concave}\\ \frac{|e_{ij}|}{|G_{i}+G_{j}|+\gamma }, & \text{otherwise}, \end{array}\right.$$ (8)

where |*e*_*ij*_| denotes the length of edge *e*_*ij*_, which is constituted by vertex *i* and *j*, *G*_*i*_ and *G*_*j*_ is the Gaussian curvature value of vertex *i* and *j* respectively. *γ* is a small constant (we choose *γ* = 0.0001 for all examples demonstrated in the manuscript) to prevent the divisor in Eq (8) to be zero. We make a large variation in field data in concave regions, which leads to a phenomenon that these concave regions can be distinguished from other areas on dental mesh, through setting small constant *β* (*β* = 0.01) when either vertex *i* or *j* lies on concave regions. Vertex *i* is treated as a concave vertex if one or more its adjacent vertexes hold the following inequality

$$\frac{\left(v_{i}-v_{adj}\right)}{|v_{i}-v_{adj}|}\cdot \left(n_{adj}-n_{i}\right)>\theta ,$$ (9)

where *v*_*adj*_ and *n*_*adj*_ is the position and normal of the vertex adjacent to the given vertex *i*, whose position and normal vector are denoted by *v*_*i*_ and *n*_*i*_ respectively, and *θ* = 0.001 is the empirical threshold value.

Besides to concavity-aware weighting scheme, the segmentation field is also related to selection of site constraints *S*, relevant contents are discussed in the following subsection.

### Site constraints in segmentation field

**Fig 2:**
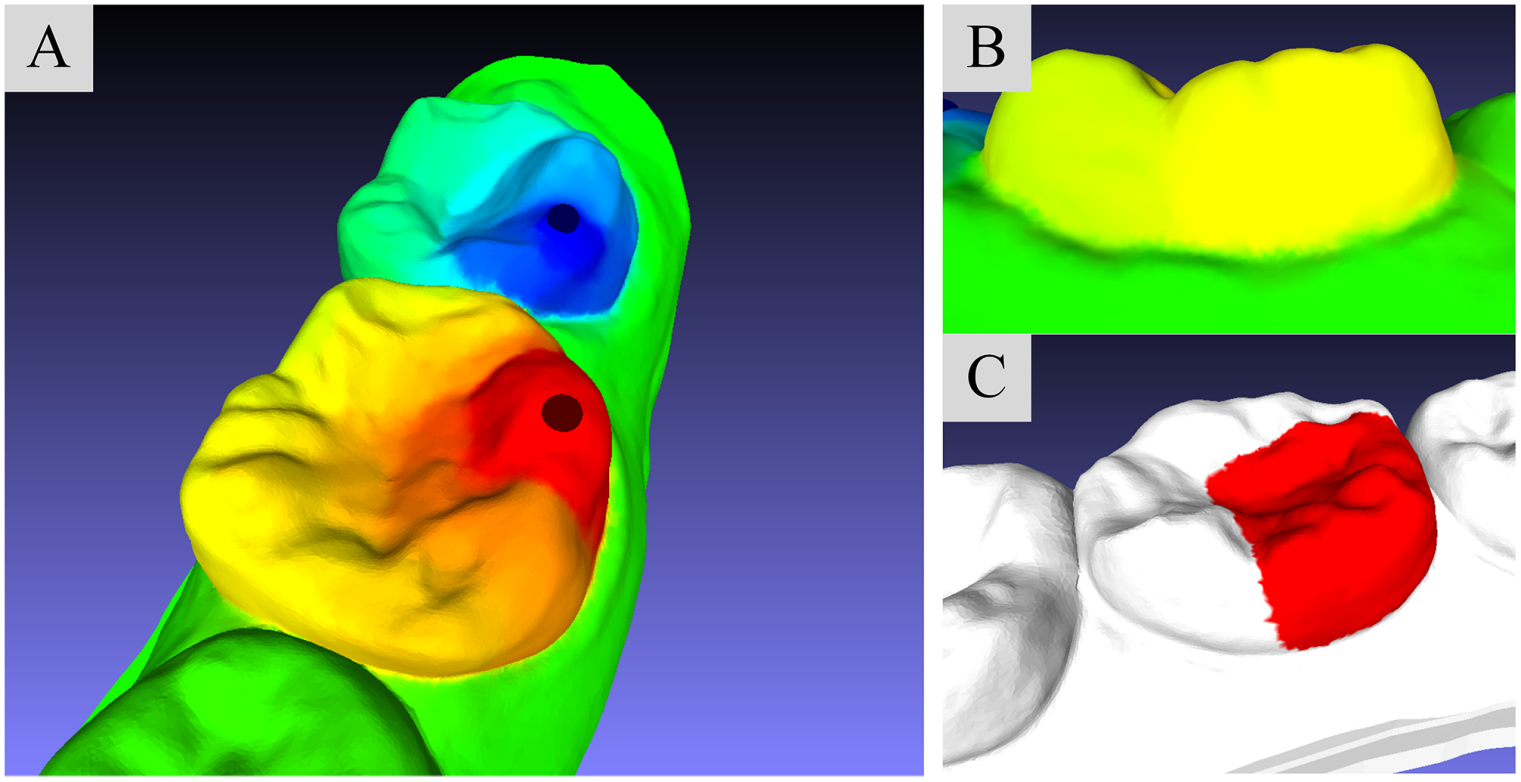
The single segmentation field with two constraint sites. (A): Visualization of the molar-target segmentation with two constraint sites. (B): Visualization of the variation of field values on the concave seam between molar and gingiva. (C): The segmentation result under a single segmentation field.

**Fig 3:**
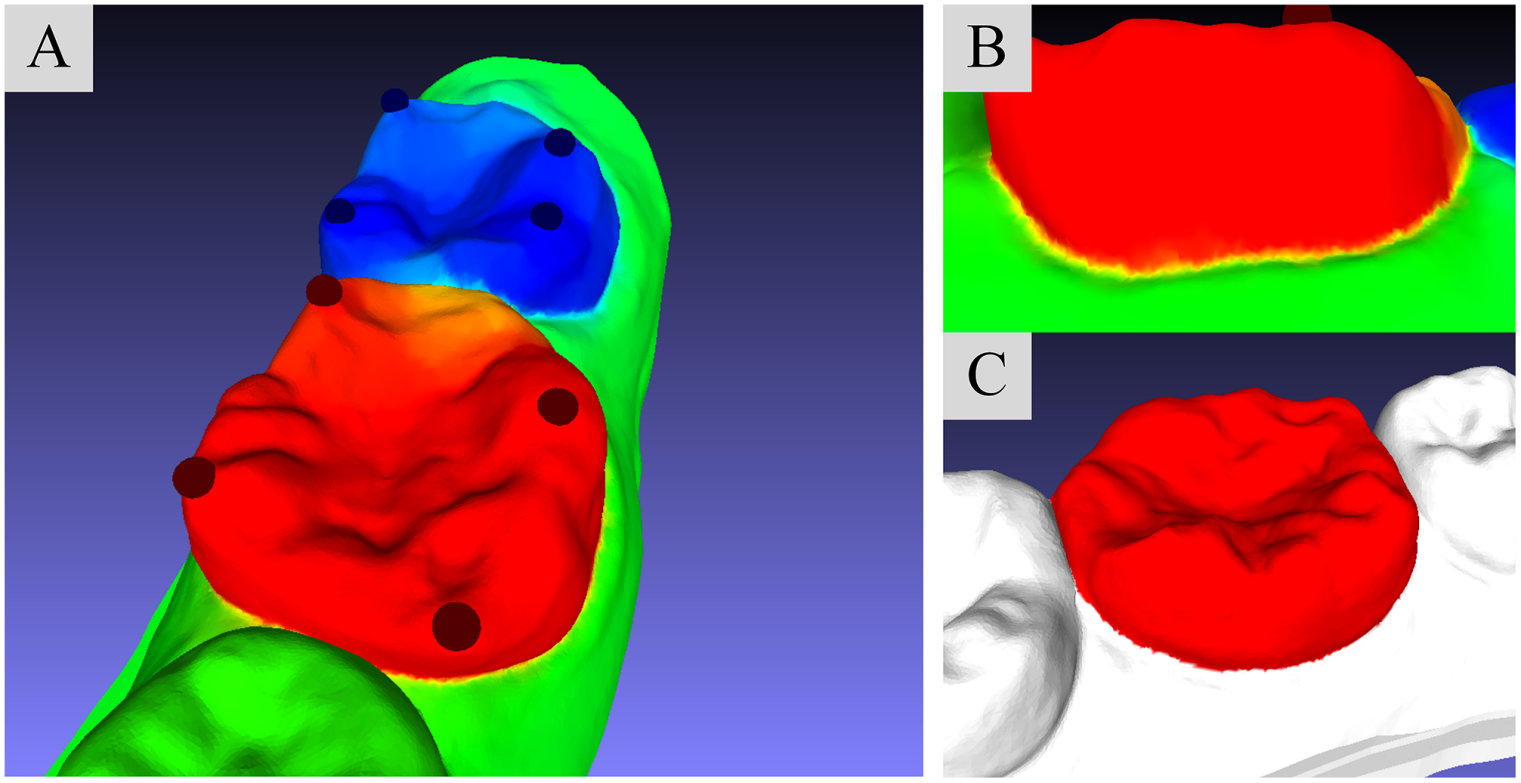
Multiple segmentation field with four constraint sites. (A): Visualization of the molar-target segmentation with four pairs of constraint sites. (B): Visualization of the variation of field values on the concave seam between molar and gingiva. (C): The segmentation result under multiple segmentation field.

As described in Eq (1), a prescribed value *s*_*i*_ of the segmentation field is assigned at site constraints *i*. At our case, the set of constraint sites are divided into two types, target set *S*_*t*_ and background set *S*_*b*_, and Eq (1) can be extended as follow:

$$u\left(i\right)=u_{i}=\left\{\begin{array}{cc} 0, & i\in S_{t}\\ 1, & i\in S_{b}. \end{array}\right.$$ (10)

where 1 and 0 are the maximum and minimum constraint values assigned to. The paths connecting maximum constraints and minimum constraints should pass through the desirable cutting boundaries. To a incisor with relevant smooth geometrical shape, a single segmentation field with two boundary constraints is sufficiently to separate from model. However, if we need to partition a molar with complex geometrical shape, such segmentation field with only two boundary constraints cannot accurately observe the candidate cutting lines. Fig 2 shows the single segmentation field with two constraint sites, which are illustrated by the red sphere (target constraint) and blue sphere (background constraint) in Fig 2A. It is clear that the color variation in concave seam, where is located between tooth and gingiva, is small, as shown in Fig 2B, so that the final segmentation result is not accurate (as illustrated in Fig 2C). In contrast, the multiple segmentation field which is constituted by four pairs of boundary constraints is able to find the concave seam we need to extract, as shown in Fig 3. Obviously, the color variation in the seam (Fig 3B) is so large that the final segmentation result (Fig 3C) is accurate. Such comparison also shows that the amount and locations of boundary constraints are critical for the accurate segmentation results.

An efficient way to identify the locations and amount of boundary constraints is to employ the method introduced by Yokesh Kumar et al. [38], in which the feature points can be automatically detected (see figure “Model with Feature Points” in Fig 1 with feature points denoted by gray spheres). By detection of tooth interstices [30], all of feature points can be grouped into *N* groups, where *N* is the amount of teeth. Hence, the feature points in same group are represented by the same color spheres (red spheres or blue spheres), as shown in figure “Grouped Feature Points” in Fig 1. With less user interaction, these processed feature points are automatically treated as the target constraints or background constraints, relevant contents are presented in the following section.

### Cutting boundary selection

**Fig 4:**
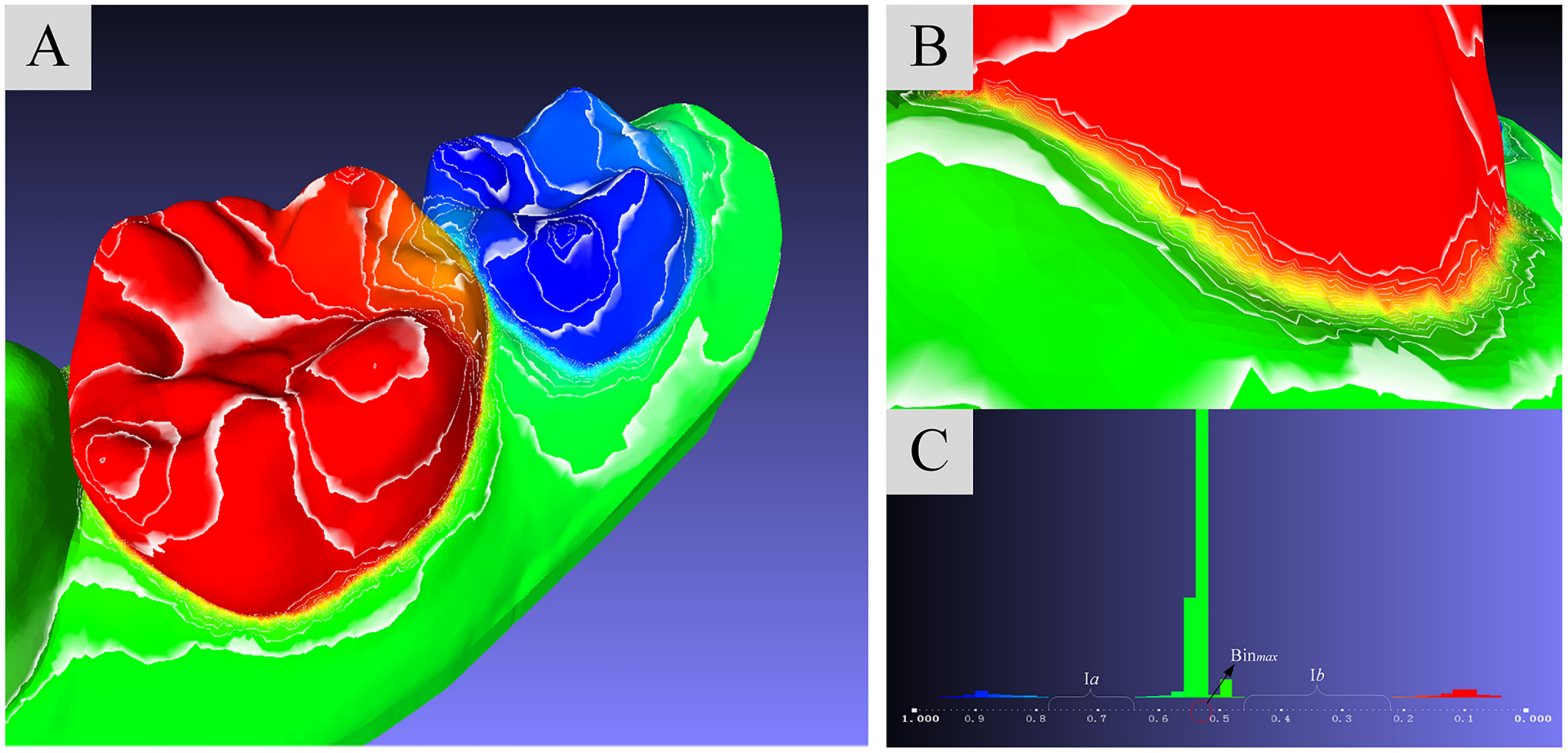
Cutting boundary selection. (A): All sampled counter lines. (B): The counter lines on concave region. (C): The histogram of vertex amount distribution on segmentation field values.

Extracting cutting boundaries in concave regions is the main purpose to segment dental model. The segmentation field has linear variation on model mesh so that a set of contour lines can be easily sampled from dental model (Fig 4A), and a large variation of field data can be detected in concave regions (Fig 4B). These properties of segmentation field provide an efficient mean to extract cutting boundaries from the set of contour linesfor teeth separation on dental model, through computing the field gradient magnitudes of these contour lines and identifying the one which possess the maximum gradient.

In Fig 4C, the histogram illustrates the distribution of the amount of mesh vertex and how many vertices fall into each small interval. As shown in the histogram, the segmentation field data are mapped into interval [0, 1]. The distribution of vertex are mainly located in three intervals, (0.04, 0.22), (0.78, 0.96) and (0.46, 0.64). The three scopes correspond to the target tooth, the adjacent tooth and the rest area on dental mesh, which are colored by red, blue and green rectangles respectively. The interval spacings, *I*_*a*_ and *I*_*b*_ in Fig 4C, between these intervals are so obviously, which means that there are fewer vertex distributing in the wider intervals and the cutting boundaries should be located in such intervals. The *Bin*_*max*_ (denoted by red circle in Fig 4C) is the bin that contains the maximum amount of vertices, thus *Bin*_*max*_ provides a median value *V*_*med*_ to split the interval [0, 1] into two intervals [0, *V*_*med*_] and (*V*_*med*_, 1]. By picking two contour lines with maximum gradient magnitudes in intervals [0, *V*_*med*_] and (*V*_*med*_, 1] respectively, the target tooth and its adjacent tooth can be easily segmented from model.

### User interface

**Fig 5:**
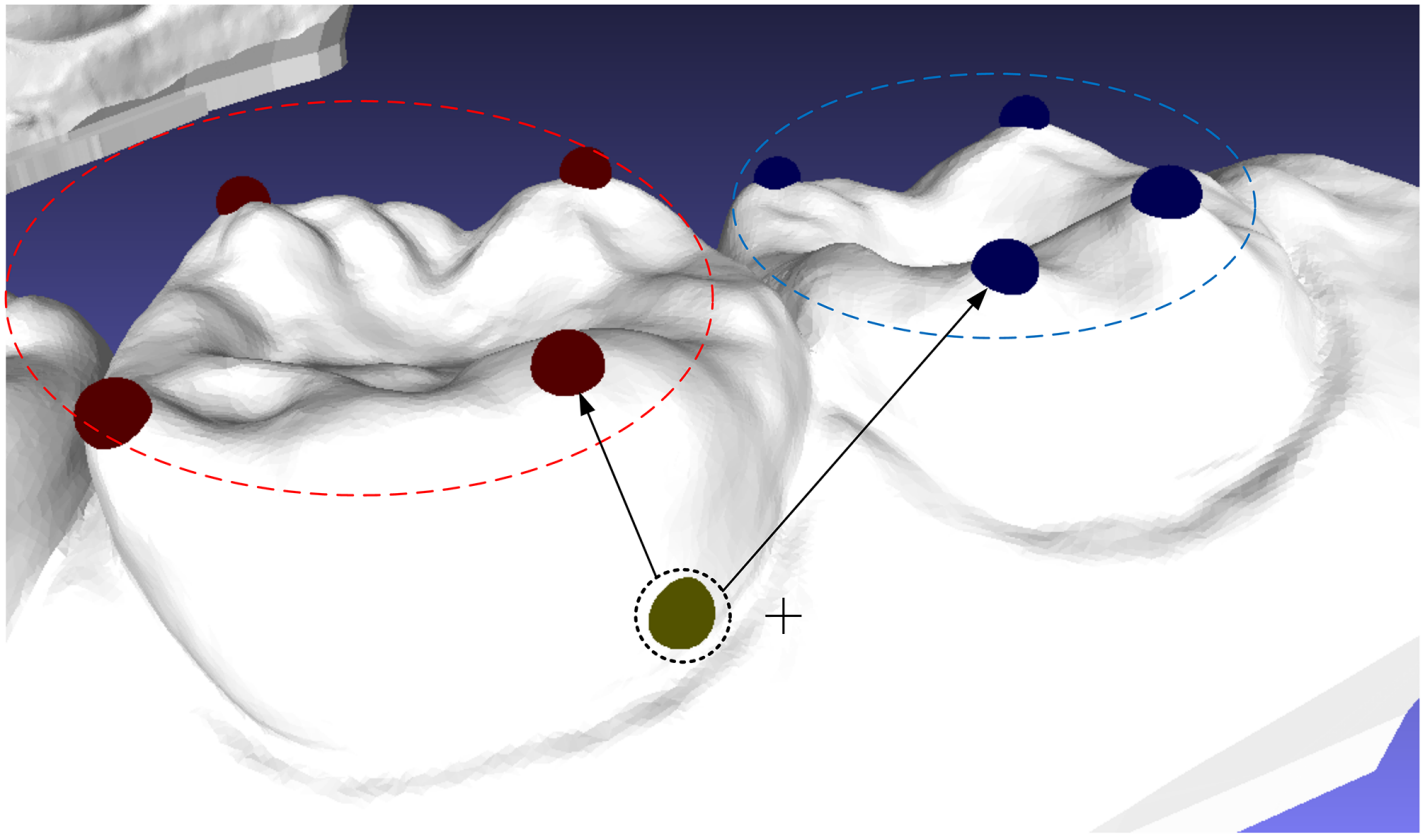
The easy-to-use interactive tool.

An easy-to-use interactive tool for dental model segmentation is indispensable in an interactive segmentation approach. In our method, teeth can effectively separated from dental model by using only a single mouse click without any additional user input. In order to indicate which tooth need to be partitioned, the interactive segmentation tool (as the cross cursor showed in Fig 5) should be placed on the target tooth. Owing to the feature points are automatically detected and grouped in advance, we only need to find the two feature point groups closet to point *p*, which is selected by user interaction tool and is denoted by yellow sphere in Fig 5), through computing the geodesic distance between point *p* and feature points (denoted by blue and red spheres in Fig 5 shows). The feature points located in the closest group are assigned as the target constraints, while the feature points belong to the next-closest one are treated as the background constraints. Thus, the target tooth and its adjacent tooth can be segmented from dental model simultaneously with two cutting contour lines. It means that we only need to take only *N*/2 times (when *N* is even) or *N*/2 + 1 times (when *N* is odd) for our interactive operation to partition all teeth from dental model, if *N* is the amount of teeth.

## Results

### Segmentation experiments

Four types of experiments were devised

- Experiments on dental models with different levels of crowding problems.
- Experiments on dental meshes with different modeling resolutions and surface noises.
- Comparative experiments conducted using a classical morphologic skeleton approach and our approach.
- Comparative experiments conducted using other common Laplacian weighting scheme and our weighting scheme.

#### Different levels of crowding problems

**Fig 6:**
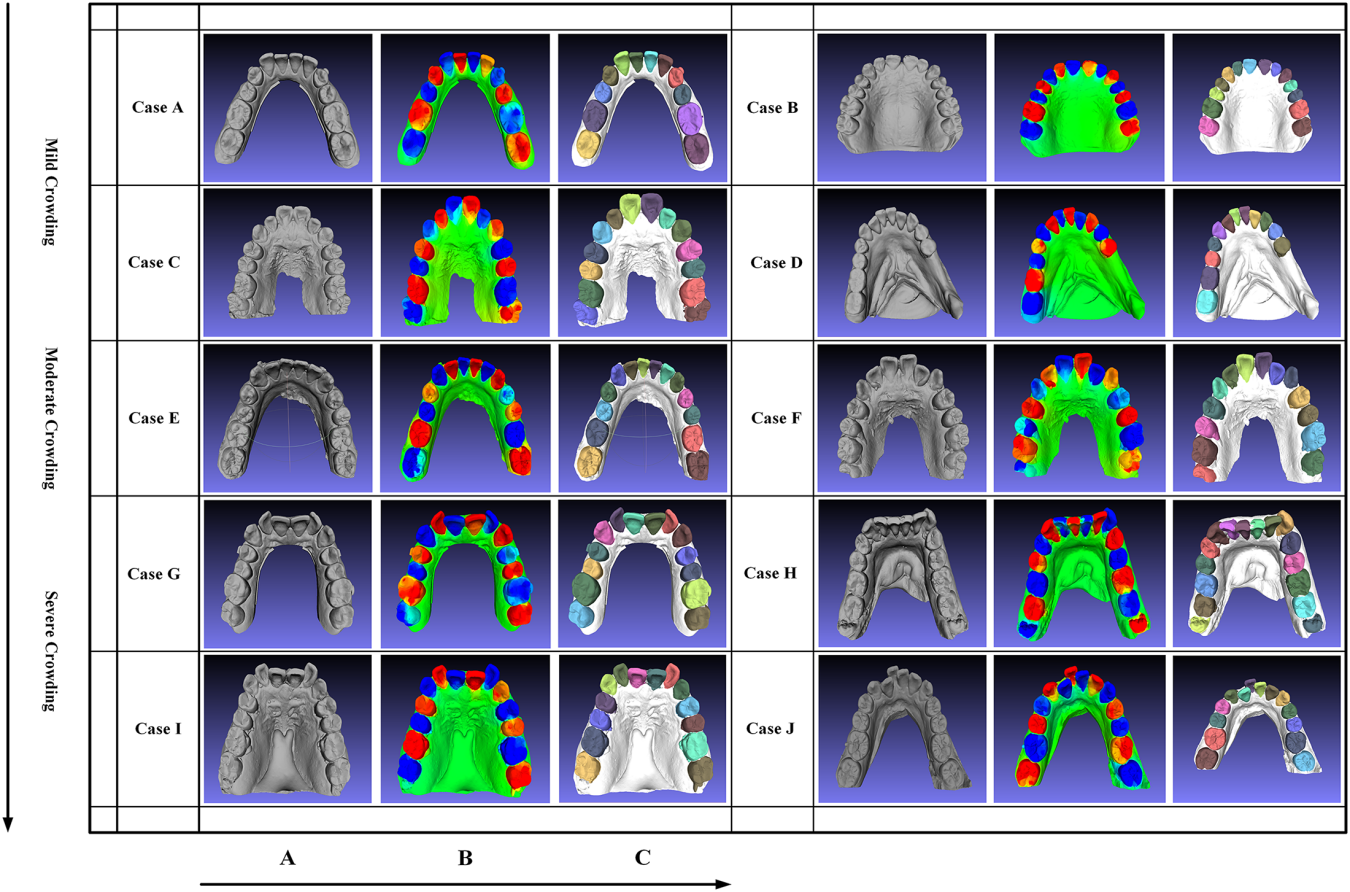
The segmentation results of employing our approach on ten dental models with crowding problems varying from “mild crowding” to “severe crowding”. (A), (B) and (C) show the consecutive segmentation operations.

To testify the effectiveness and accuracy of our method, ten dental models, including five maxilla models (B, C, F, G, I) and five mandible models (A, D, E, H, J), are segmented, which are illustrated by Fig 6. In these ten dental models, case A and G, as well as case B and D are obtained from the Internet, and the rest of dental models are obtained from patients who need medical treatments with the same scan device. According to the crowding levels, these dental models are classified into three types, including “mild crowding”, “moderate crowding” and “severe crowding”, as Fig 6 shows. Since these dental models are collected from different sources, the scales and qualities of models are different, which can be described in Table 1.

**Table 1:** The scale statistic for all models illustrated in Fig 6.

Model Case	A	B	C	D	E	F	G	H	I	J
**Size**	14,750 KB	11,077 KB	44,104 KB	22,093 KB	38,991 KB	49,949 KB	14,752 KB	52,498 KB	55,917 KB	32,708 KB
**Vertices**	151022	113489	451622	226232	399264	511478	151057	537571	572587	334925
**Triangles**	302118	226843	903240	452460	798524	1022952	302118	1075146	1145170	669852

#### Different levels of modeling resolutions and surface noises

**Fig 7:**
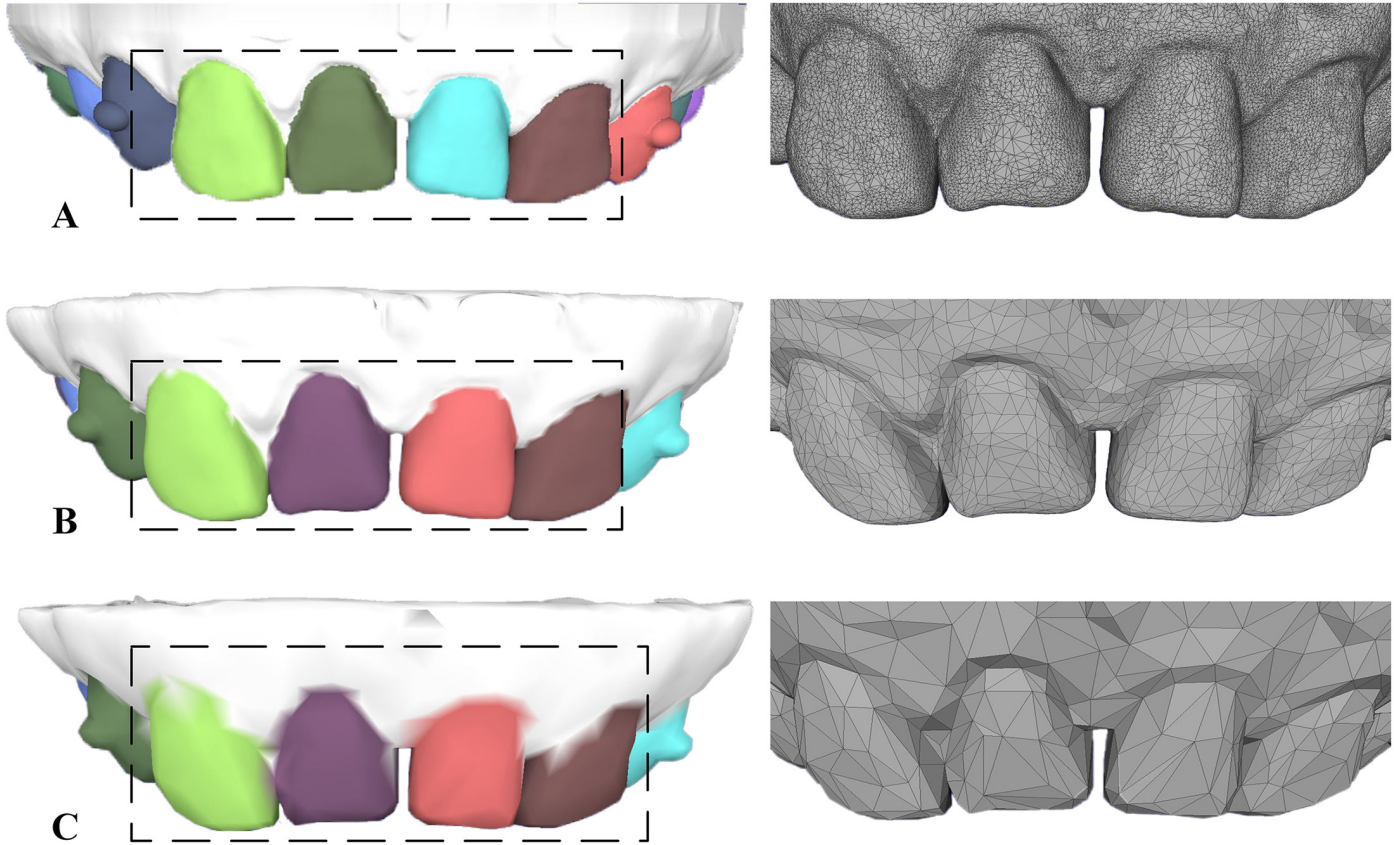
The segmentation results of employing our approach on three dental models with different surface resolutions. From up to down ((A) to (C)), each dental model is with 301964, 50000 and 10000 faces, respectively.

**Fig 8:**
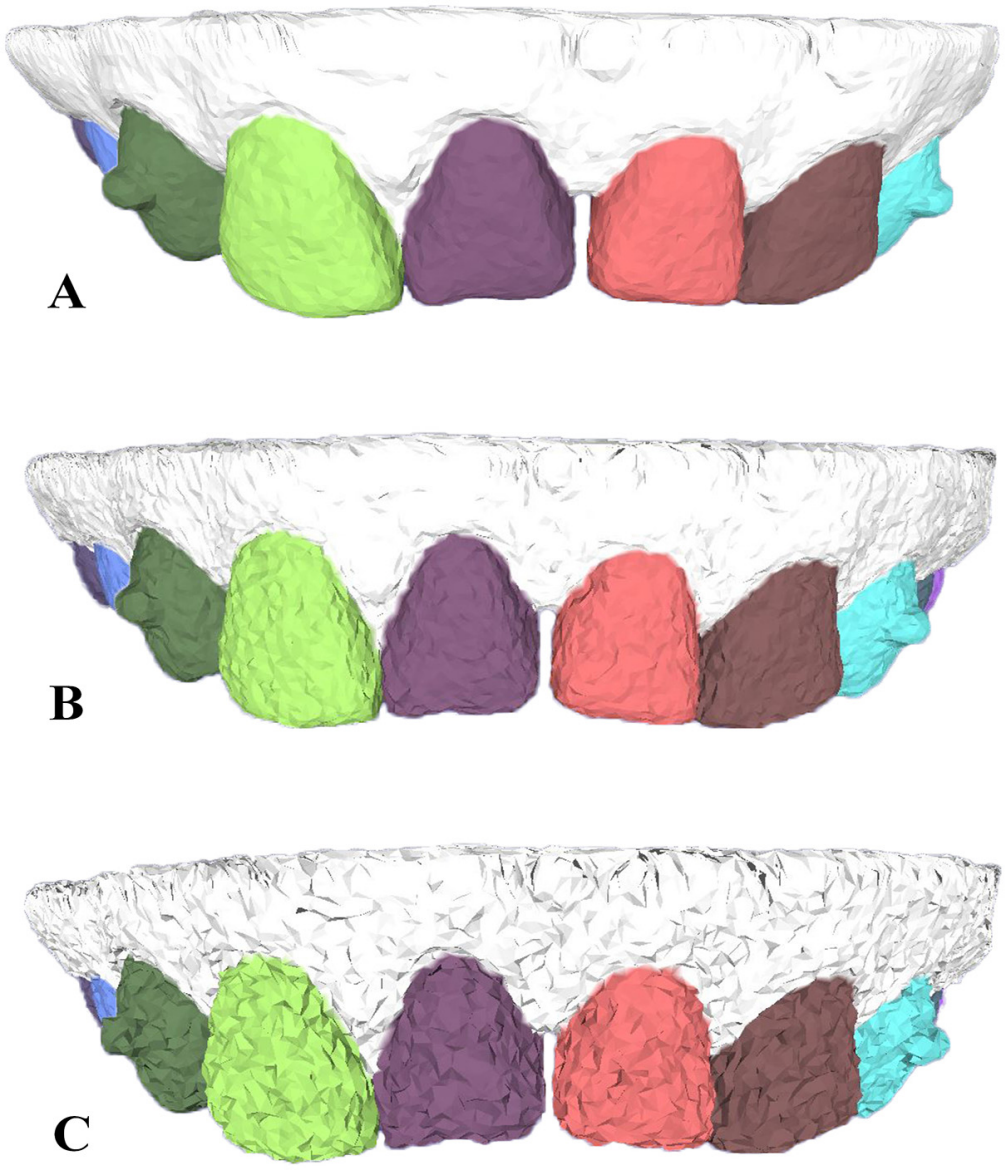
The segmentation results of employing our approach on three dental models with different noises. From up to down ((A) to (C)), the dental model is coupled with 0.05, 0.2 and 0.5 mean edge-length Gaussian noise, respectively.

The segmentation field in our method is sensitive to concave regions and is stable in the presence of different levels of tessellations and mesh noises. In Fig 7, we tested three dental models with different mesh resolutions and found that our method is largely sensitive to different tessellations on dental models, even to coarse tessellation as illustrated in Fig 7C. In addition, our dental model segmentation method is also insensitive to surface noises of dental models, and similar segmentation results are obtained under different scales of mesh noises in Fig 8.

#### Comparison with classical morphologic skeleton segmentation approach

**Fig 9:**
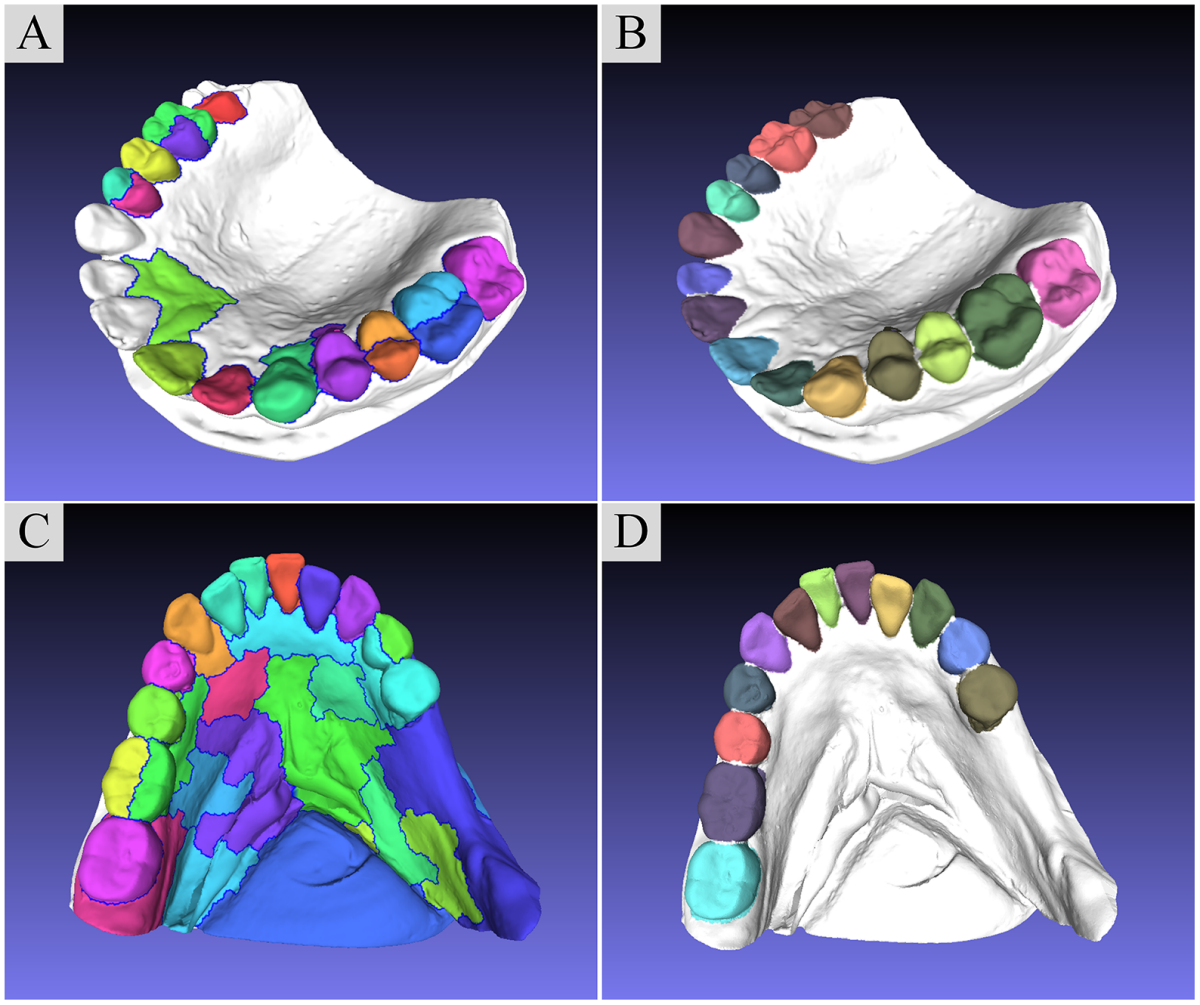
The comparison between our method and the segmentation algorithm presented by [28]. (A) and (C) are the segmentation result of [28] with model *case B* and *case D* in Fig 6, and (B) and (D) are the correspond segmentation results of our method. The average time consumption of single tooth separation in A and C were 42629*ms* and 159208*ms*, and the correspond time consumption in B and D were 1319*ms* and 3279*ms*, respectively.

By running source code that is available from [28], a classical morphologic skeleton segmentation approach can be used to compare with our approach. Under the exactly same experimental conditions with same dental models, the comparison of segmentation results are shown in Fig 9. Obviously, the striking failures, including “under-segmentation” and “over-segmentation” in Fig 9A and 9C, are avoided in our method as show in Fig 9B and 9D. On the other hand, a huge difference in time consumption between segmentation method of [28] and ours are also be detected in the comparison results. The experimental results, as shown in Fig 9A and 9C, cast 42629*ms* and 159208*ms* respectively, under framework adopted in [28], while the experimental results, as shown in Fig 9B and 9D, cast 1319*ms* and 3279*ms* respectively, under our segmentation framework. Overall, the difference between method of [28] and ours in errors and time consumption are closely related to the curvature threshold value issue. In [28], the curvature threshold value is automatically adjusted, through comparing the amount of segment parts and number of tooth that user entered for equality. Such mechanism is very time-costing due to the fact that, in order to find a satisfied threshold value, algorithm need to traverse whole the mesh vertex set multiple times with complex computation process. Conversely, corresponding iteration traversal times in our method is limited. The segmentation field computation, which is the most time consuming operation in the procedure of our segmentation framework, only need to traverse mesh vertices once. In addition to this, the appropriate curvature threshold values are difficult to obtain in some cases, the segmentation failures occurred in Fig 9A and 9C are resulted from the inappropriate threshold values. But, the threshold selection can be avoided in our method, such advantage is a significant property to reflect the superiority of our segmentation method, as the comparisons shown.

#### Comparison with other Laplacian weighting scheme

**Fig 10:**
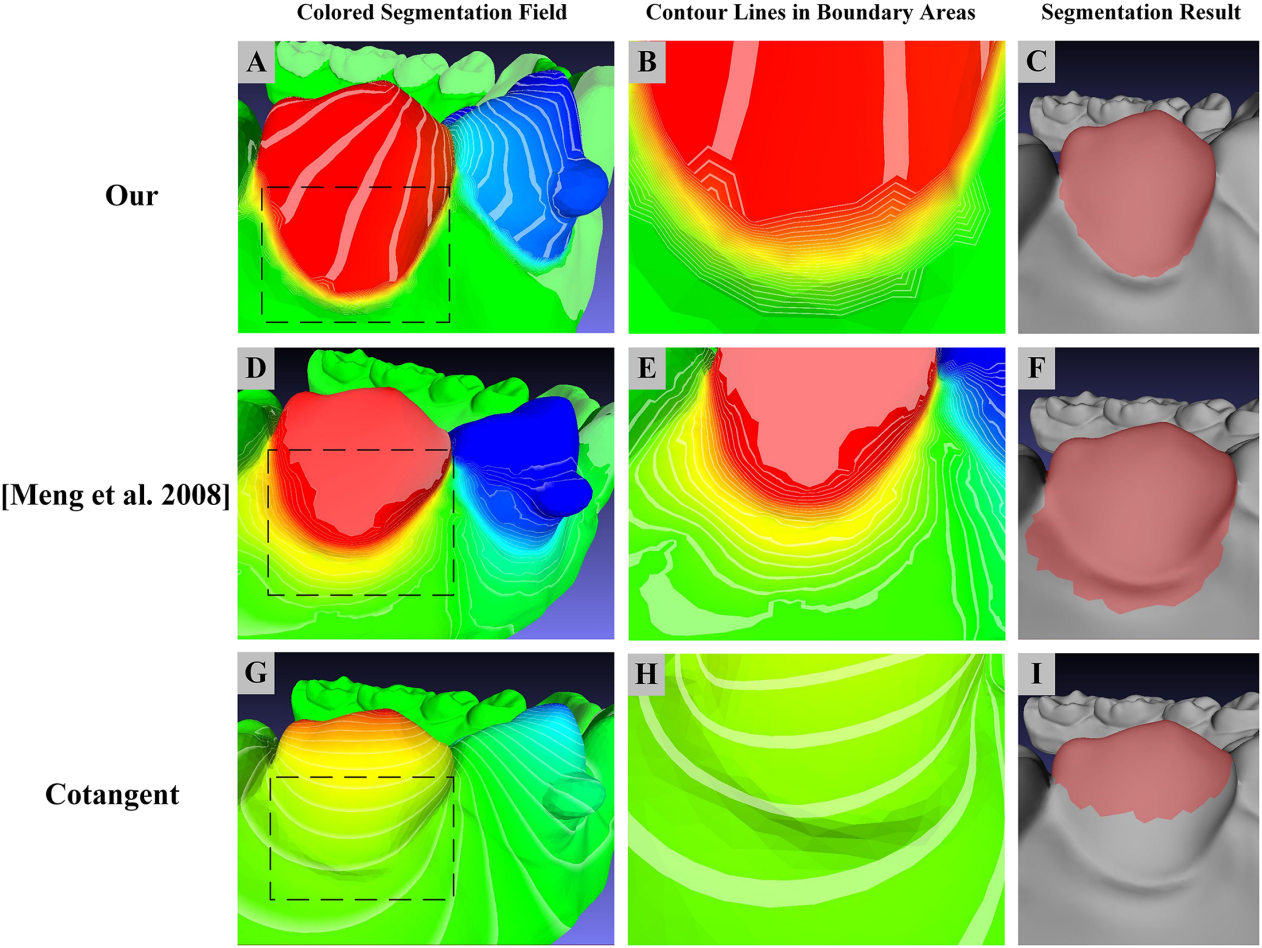
The concavity-sensitive weighting scheme produces more denser contour lines at tooth boundary areas compared with other weighting schemes, hence leads to better segmentation result. (**Left**)((A), (D) and (G)) are segmentation field, (**Middle**)((B), (E) and (H)) are detailed contour lines in tooth boundary ares, (**Right**)((C), (F) and (I)) are relevant segmentation results under different weighting schemes.

The other common Laplacian weighting schemes [39, 40], are implemented and tested with our segmentation field framework. In Fig 10, we can see that, compare with the classical cotangent weighting scheme [39](Fig 10G and 10H),a large color variation can be obviously detected in the concave seams of dental model under the weighting scheme in our method (Fig 10A and 10B) and the weighting scheme adopted by [40] (Fig 10D and 10E). In addition, the concavity-sensitive weighting scheme make the field value variation mainly gathered at narrower concave areas with denser contour lines, as illustrated in Fig 10B, compare to the weighting scheme adopted by [40] (Fig 10E), leading to better segmentation result (Fig 10C and 10F).

### Evaluation

**Fig 11:**
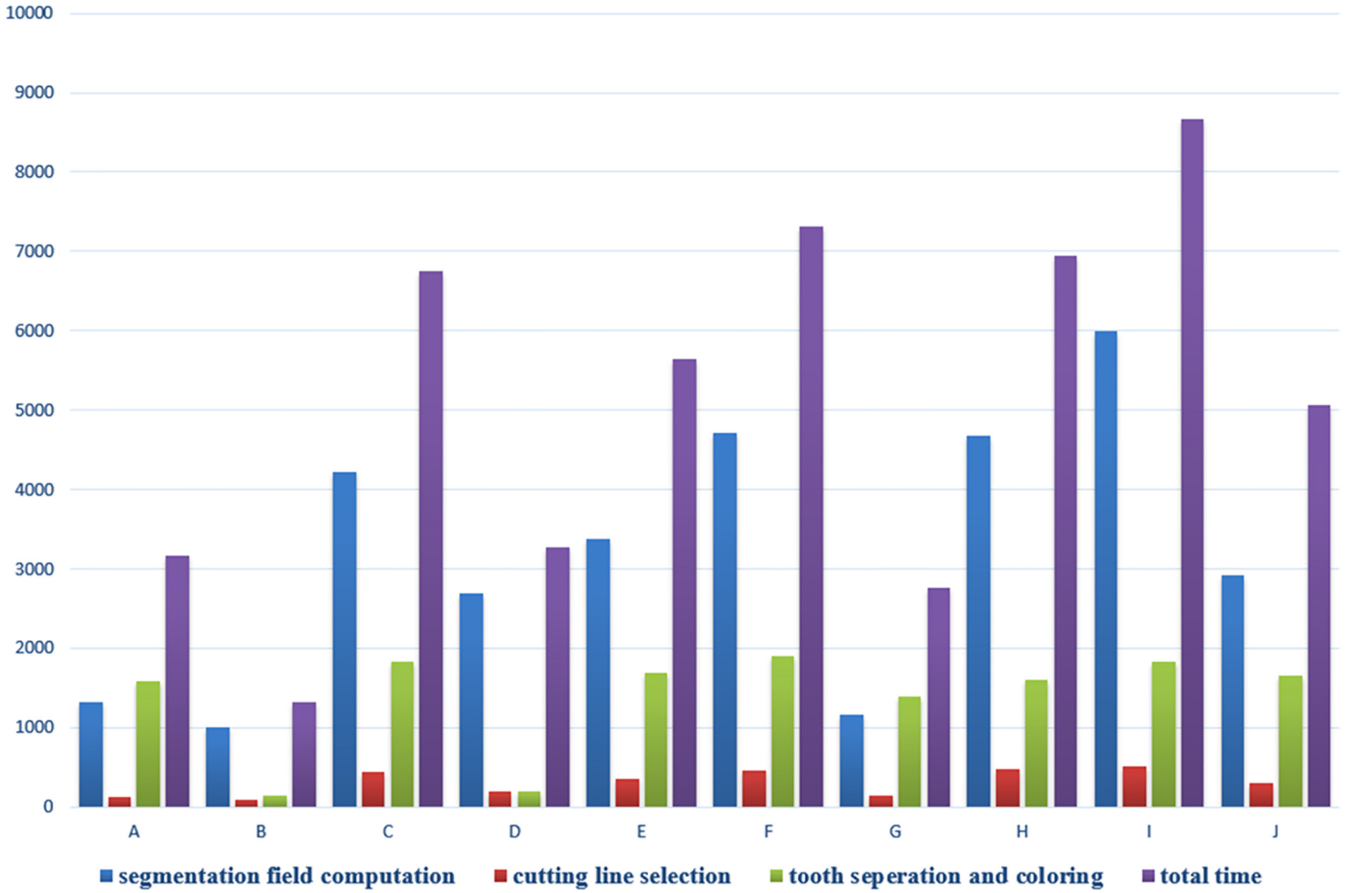
Average time consumption of single tooth segmentation in experiments illustrated in Fig 6. Blue, red, green and purple lines indicate the time consumed by segmentation field computation, cutting line selection, tooth separation and coloring and total time, respectively (in *ms*).

The time consumption of our method on the ten dental models in Fig 6 was recorded. In Fig 11, the blue, red, green and purple lines indicate the time consumed by segmentation field computation, cutting line selection, tooth separation and coloring and total time respectively. The most time-consuming operations are segmentation field computation, while the least one are cutting line selection in our method. The relevant model scales are listed in Table 1. Our segmentation method is implemented as a C plus plus plug-in for Meshlab [41], a open source system for the processing and editing of three dimensional triangular meshes, with Intel Core i5-4200H CPU @2.8G Hz and 4GB RAM.

## Conclusion

In this article, a novel, effective and efficient dental-target segmentation approach is presented. A segmentation field which is sensitive to concave seams of dental model is computed, through less user interaction to assign constraint sites. The cutting boundaries to separate target tooth from model mesh can be directly extracted from the smooth scalar segmentation field. As shown in the experiment results and comparisons, the properties of our approach is able to present segmentation results with less errors and less time consumption, regardless of different levels of crowding problems and different model qualities. Our future work will involve avoiding user interaction to develop a fully automatic segmentation algorithm, as well as speeding up the operation of our algorithm through optimization algorithm and applying parallel computing technique.
